# Supplementary material for: Nucleosome wrapping states encode principles of 3D genome organization
Source: Nat Commun. 2025 Jan 3;16:352. doi: 10.1038/s41467-024-54735-8 (PMC11699143; doi:10.1038/s41467-024-54735-8)
Supplement: Supplementary file 1 — Supplementary Information [file 41467_2024_54735_MOESM1_ESM.pdf]

# Nucleosome wrapping states encode principles of 3D genome organization

Zengqi Wen[1]✉, Ruixin Fang[2], Ruxin Zhang[2], Xinqian Yu[2], Fanli Zhou[1] and Haizhen Long[2]✉

[1]School of Medicine, Shenzhen Campus of Sun Yat-Sen University, Sun Yat-Sen University, Shenzhen, Guangdong, 518107, China. [2]Institute of Molecular Physiology, Shenzhen Bay Laboratory, Shenzhen, 518132, China. ✉e-mail: wenzq7@mail.sysu.edu.cn; longhaizhen@szbl.ac.cn

## Supplementary Information:

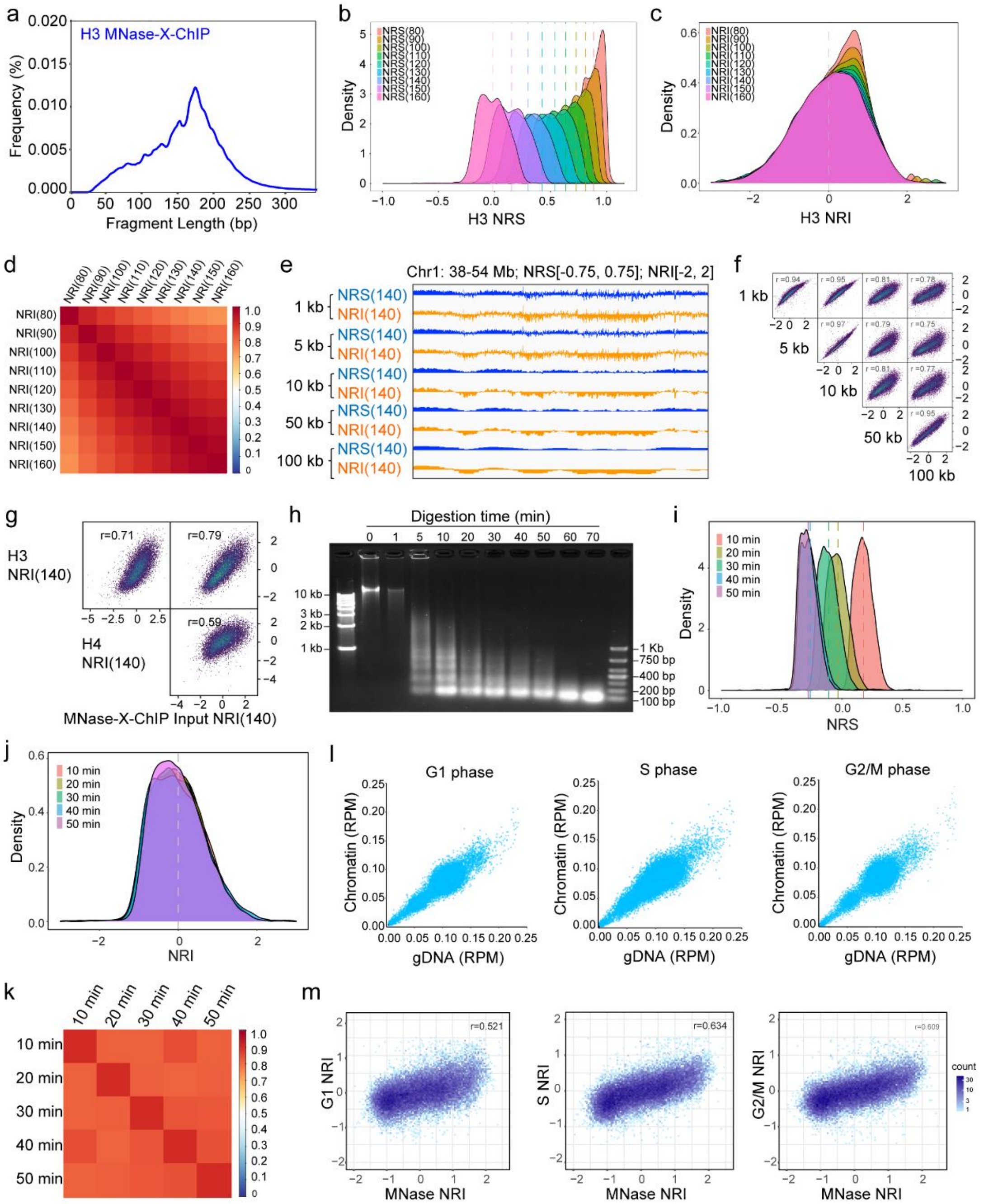


**Figure S1. Nucleosome wrapping index is robust for nucleosome wrapping state characterization**

a, Histogram shows the length distribution of DNA fragments enriched by histone H3 within 0-350 bp range. b-c, Histograms show the distribution of genome-wide NRSs (b) or NRIs (c) when different break point is chosen as in Fig. 1b. d, Heatmap shows the correlations between genome-wide NRIs counted with 100 Kb bins (n = 27268) when break point is chosen as in Fig. 1b. e, IGV tracks show NRSs and NRIs of H3 on Chromosome 1, calculated using different bin sizes. The signal ranges are indicated at the top of figure. f, Dot plots show the genome-wide correlations between NRIs of H3 calculated using different bin sizes, counted with 100 Kb bins (n = 27268). g, Dot plots show the genome-wide correlations between NRI (140)s of

H3, H4 or MNase-X-ChIP input, counted with 100 Kb bins (n = 27268). h, Agarose gel shows the products of DNA after time-course digestion by MNase. This experiment was repeated twice with similar results. i-j, Histograms show the distribution of genome-wide NRSs (i) or NRIs (j) under time-course MNase digestion, counted with 100 Kb bins (n = 27268). k, Heatmap shows the genome-wide correlation between MNase NRIs of under time-course digestion, counted with 100 Kb bins (n = 27268). l, Dot plots show the correlations between genome coverage of sonicated genomic DNA and MNase digested chromatin in G1, S and G2/M cells, counted with 100 Kb bins (n = 27268). m, Dot plots show the correlation between MNase NRI of cell population and MNase NRI of G1, S or G2/M cells, counted with 100 Kb bins (n = 27268). “r” indicates the Pearson correlation coefficient. Source data are provided as a Source Data file.

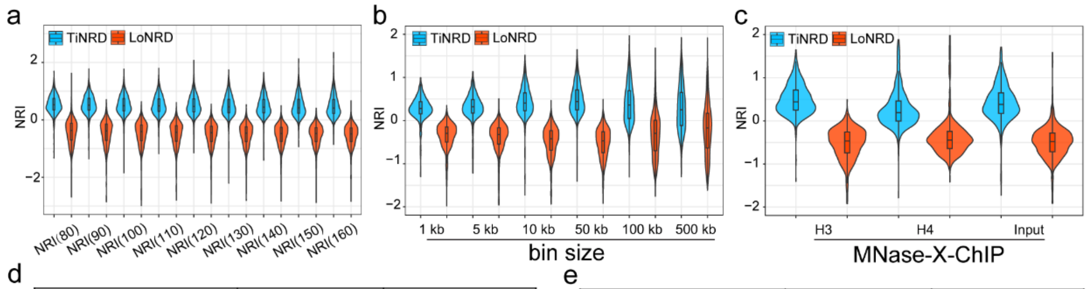


d

| *S. cerevisiae* | LoNRD | TiNRD |
|---|---|---|
| Number | 1027 | 1051 |
| Median size (Kb) | 5 | 5 |
| Coverage (%) | 49.4 | 50.6 |

e

| *D. melagaster* | LoNRD | TiNRD |
|---|---|---|
| Number | 836 | 804 |
| Median size (Kb) | 60 | 70 |
| Coverage (%) | 46.2 | 53.8 |

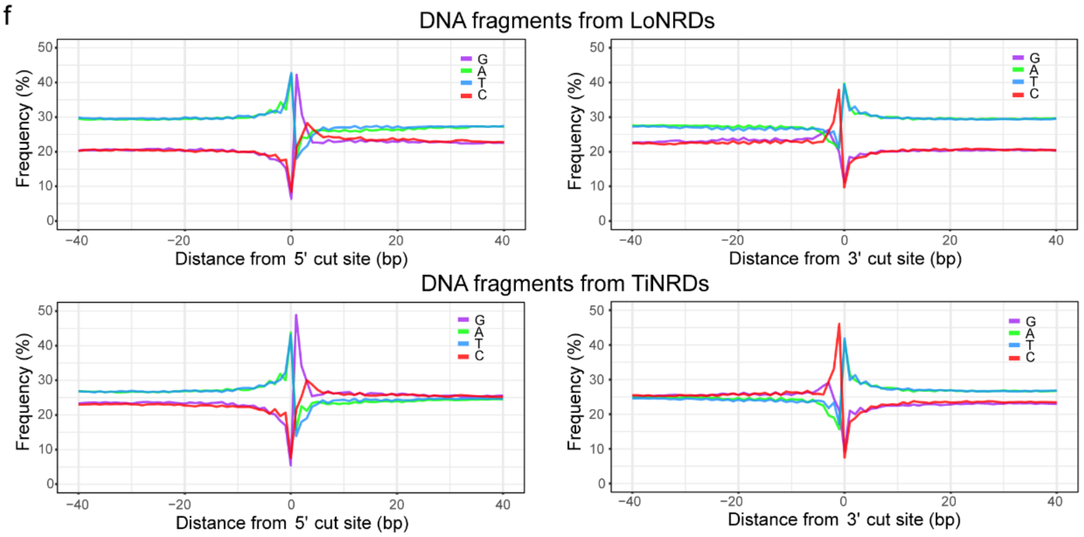


**Figure S2. Nucleosome wrapping domain is detected in yeast, fly and mouse genome**

a-c, Violin plots show the characteristic distribution of genome-wide NRIs in LoNRDs (n = 723) and TiNRDs (n = 725). NRIs were calculated with different break points (a), bin sizes (b) or dataset (c). The center line and box limits of boxplots represent median, first and third quantile. d-e, Tables show the number, median length and genome coverage of TiNRDs and LoNRDs of *S. cerevisiae* (d) and *D. melanogaster* genome (e). f, Meta-profiles show nucleotide

frequency around the 5' or 3' cut sites of DNA fragment mapped within LoNRDs and TiNRDs. Source data are provided as a Source Data file.

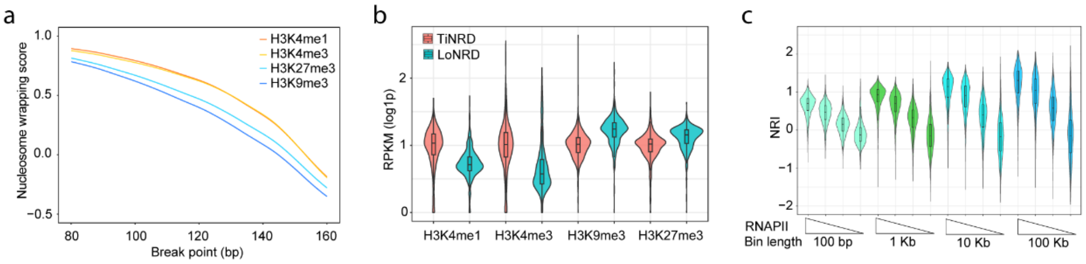


**Figure S3. Nucleosomes wrap tighter in euchromatin than in heterochromatin**

a, Line plot shows the H3 NRS(140)s within H3K4me1, H3K4me3, H3K9me3 and H3K27me3 peak regions, calculated with H3 enriched DNA fragments in the corresponding peak regions, with break points range from 80-160 bp with 1 base pair step. b, Violin plots show the distribution of H3 normalized H3K4me1, H3K4me3, H3K9me3 and H3K27me3 signals in LoNRDs (n = 723) and TiNRDs (n = 725). c, Violin plots show H3 NRIs in gene body regions (n = 43511) grouped descendently by Polr2a signal. H3 NRIs calculated with 100 bp, 1 Kb, 10 Kb and 100 Kb bin resolution. The center line and box limits of boxplots represent median, first and third quantile. Source data are provided as a Source Data file.

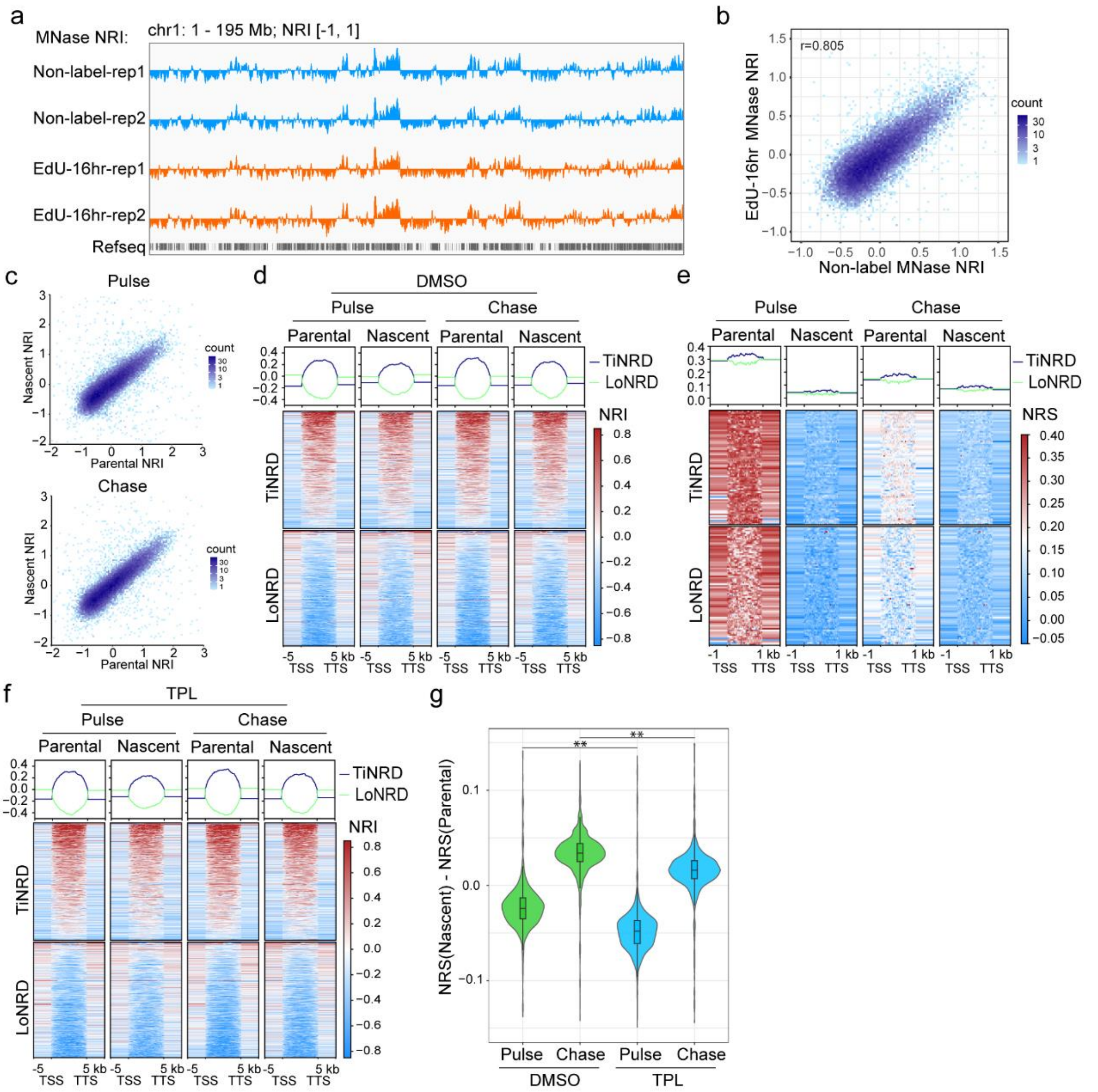


**Figure S4. Transcription promotes nascent nucleosome wrapping**

a, IGV tracks show the pattern of MNase NRI of non-label cells and EdU-labeled cells. EdU was labeled for 16 hours. Two replicates were shown for each culture condition. The signal ranges are indicated at the top of figure. b, Dot plot shows the correlation between MNase NRI of non-label cells and EdU-labeled cells, counted with 100 Kb bins (n = 27268). c, Dot plots show the genome-wide correlation between MNase NRI(140) of parental and nascent samples, under “Pulse” and “Chase” conditions, counted within NRDs (n = 1448). MNase NRI(140) was calculated using xMNase-seq dataset with 100 kb bins and break point at 140 bp. d, Heatmaps show MNase NRI(140) of parental and nascent nucleosomes from “Pulse” and “Chase” conditions, within LoNRDs (n = 723) and TiNRDs (n = 725). e, Heatmaps show MNase NRS(140) of parental and nascent nucleosomes from pulse and chase conditions, re-analyzed of MINCE-seq dataset[16]. f, Heatmaps show NRI of parental and nascent nucleosomes after TPL treatment, within LoNRDs (n = 723) and TiNRDs (n = 725). g, Violin plots show the dynamics of nucleosome wrapping after DNA replication, with or without TPL treatment. The difference of NRS was calculated by subtracting the MNase NRS(140) of parental nucleosome from the MNase NRS(140) of nascent nucleosome in each NRD (n = 1448). The center line and box limits of boxplots represent median, first and third quantile. Two tail Student's t-test

was used, ** indicates $P < 0.01$. Source data are provided as a Source Data file.

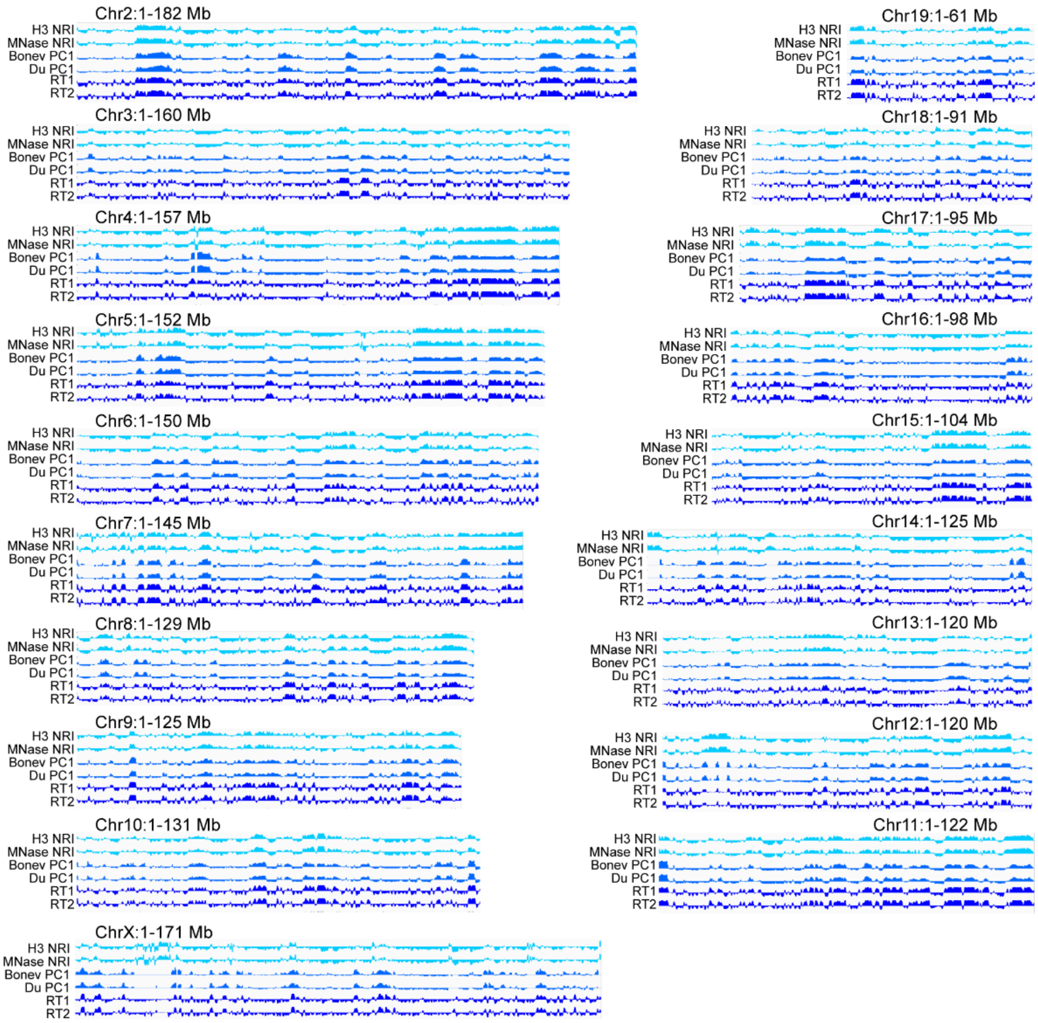


**Figure S5. Nucleosome unwrapping states delineate 3-D genome organization**

a, IGV tracks show per chromosome distribution of H3 NRI(140), MNase NRI(140), Hi-C PC1 values and replication timing values, except for chromosome Y. Chromosome 1 is shown in Fig. 5a.

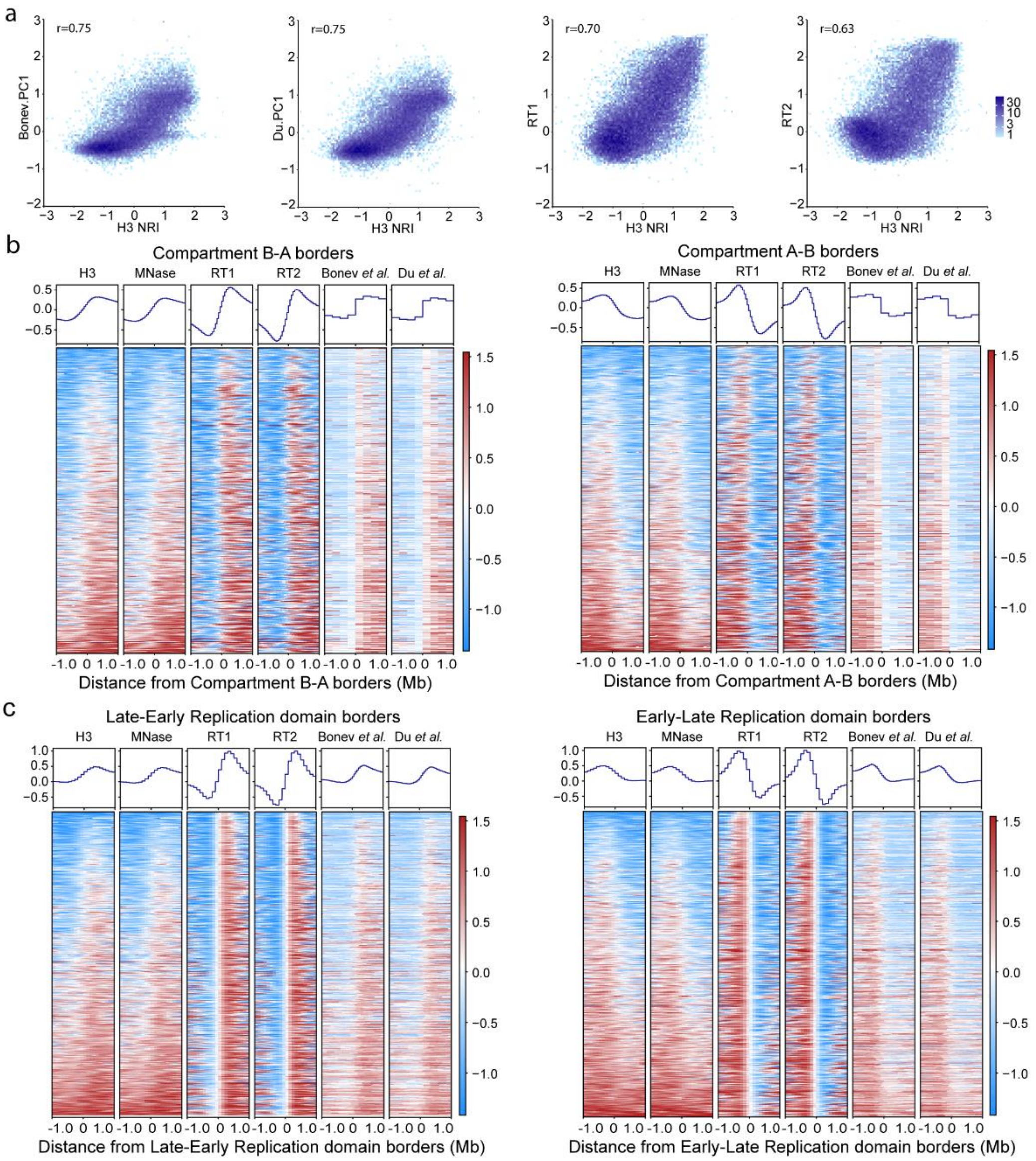


**Figure S6. Nucleosome unwrapping domains delineate Hi-C compartment domains and replication timing domains.**

a, Dot plots show the genome-wide correlations between H3 NRI(140) and Hi-C PC1 values or replication timing values, counted with 100 Kb bins (n = 27268). “r” indicates the Pearson correlation coefficient. b-c, Heatmaps show the distribution of H3 NRI(140), MNase NRI(140), Hi-C PC1 values and replication timing values on the 2 Mb regions around B-A compartment domain borders (n = 637) (left panel) or A-B compartment domain borders (n = 637) (right panel) (b), or on the 2 Mb regions around late-early RT domain borders (n = 841) (left panel) or early-late RT domain borders (n = 841) (right panel) (c). Source data are provided as a Source Data file.

## Source Data files

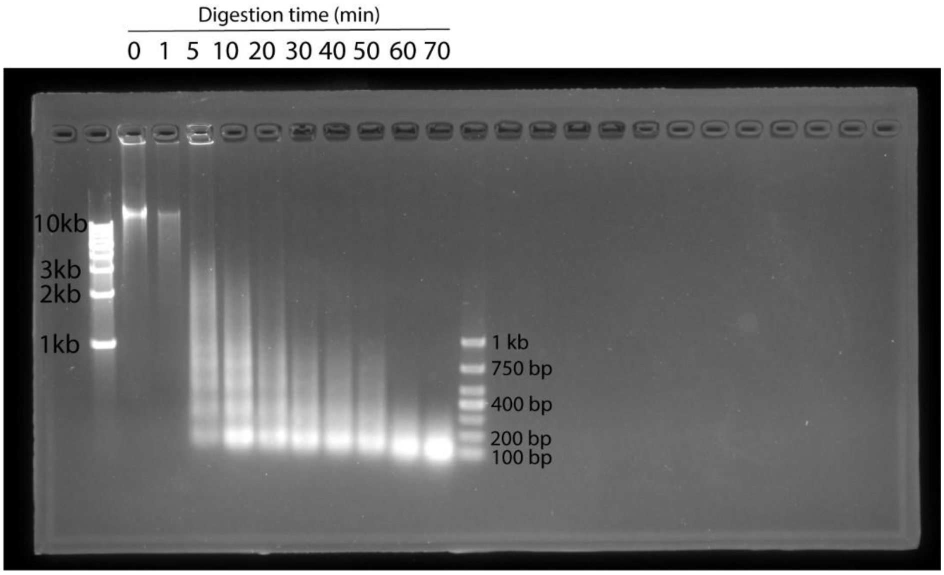


**Source Data file 1: uncropped gel of supplementary figure 1h.**
