# Supplementary material for: Nucleosome wrapping states encode principles of 3D genome organization
Source: Nat Commun. 2025 Jan 3;16:352. doi: 10.1038/s41467-024-54735-8 (PMC11699143; doi:10.1038/s41467-024-54735-8)
Supplement: Supplementary file 2 — Reporting Summary [file 41467_2024_54735_MOESM2_ESM.pdf]

nature portfolio

Corresponding author(s): Zengqi Wen

Last updated by author(s): Oct 28, 2024

Reporting Summary

Nature Portfolio wishes to improve the reproducibility of the work that we publish. This form provides structure for consistency and transparency in reporting. For further information on Nature Portfolio policies, see our Editorial Policies and the Editorial Policy Checklist.

Statistics

For all statistical analyses, confirm that the following items are present in the figure legend, table legend, main text, or Methods section.

n/a	Confirmed	
☐	☒	The exact sample size (n) for each experimental group/condition, given as a discrete number and unit of measurement
☐	☒	A statement on whether measurements were taken from distinct samples or whether the same sample was measured repeatedly
☐	☒	The statistical test(s) used AND whether they are one- or two-sided<br>*Only common tests should be described solely by name; describe more complex techniques in the Methods section.*
☒	☐	A description of all covariates tested
☒	☐	A description of any assumptions or corrections, such as tests of normality and adjustment for multiple comparisons
☐	☒	A full description of the statistical parameters including central tendency (e.g. means) or other basic estimates (e.g. regression coefficient) AND variation (e.g. standard deviation) or associated estimates of uncertainty (e.g. confidence intervals)
☐	☒	For null hypothesis testing, the test statistic (e.g. F, t, r) with confidence intervals, effect sizes, degrees of freedom and P value noted<br>*Give P values as exact values whenever suitable.*
☒	☐	For Bayesian analysis, information on the choice of priors and Markov chain Monte Carlo settings
☒	☐	For hierarchical and complex designs, identification of the appropriate level for tests and full reporting of outcomes
☐	☒	Estimates of effect sizes (e.g. Cohen's d, Pearson's r), indicating how they were calculated

Our web collection on statistics for biologists contains articles on many of the points above.

Software and code

Policy information about availability of computer code

Data collection	Wrapping-seq data and E/L replication sequencing (Repli-seq) data were collected by DNBSEQ-T7 platforms.
Data analysis	1. Wrapping-seq data and E/L replication sequencing (Repli-seq) data were analysed by Cutadapt (version 4.3), Bowtie2 (version 2.5.1), picard MarkDuplicates (version v2.27.5), samtools (version 1.17), bedtools (version 2.30.0), Homer2, Deeptools (version 3.5.1), R (version 4.0.2) and IGV(Integrative Genomics Viewer) (version 2.8.9).<br>2. The Jupyter notebook used to calculate nucleosome wrapping score and nucleosome wrapping index can be found at https://github.com/WenZengqi/nucleosome-wrapping-index and archived in Zenodo (https://doi.org/10.5281/zenodo.14000607).

For manuscripts utilizing custom algorithms or software that are central to the research but not yet described in published literature, software must be made available to editors and reviewers. We strongly encourage code deposition in a community repository (e.g. GitHub). See the Nature Portfolio guidelines for submitting code & software for further information.

Data

Policy information about availability of data

All manuscripts must include a data availability statement. This statement should provide the following information, where applicable:

- Accession codes, unique identifiers, or web links for publicly available datasets
- A description of any restrictions on data availability
- For clinical datasets or third party data, please ensure that the statement adheres to our policy

High throughput sequencing data generated in this study were deposited to Gene Expression Omnibus (GEO) under accession GSE243091.
Drosophila MINCE-seq fastq data was downloaded according to GEO record GSE76120, and mapped to drosophila genome version dmel-r6.16 (https://flybase.org). Samples GSM1974516 and GSM1974518 were merged as nascent input, and used for NRI visualization and NRD detection with 100 kb bin resolution as shown in Fig. 2d. Samples GSM1974517 and GSM1974519 were merged as nascent pulldown. Samples GSM1974528 and GSM1974530 were merged as chase input. Samples GSM1974529 and GSM1974531 were merged as chase pulldown. NRS was calculated for nascent input, nascent pulldown, chase input and chase pulldown samples as shown in Fig. S4e. S. cerevisiae MNase-seq fastq data was downloaded according to GEO record GSE30551, and mapped to yeast genome GCF_000146045.2 (https://www.ncbi.nlm.nih.gov). Sample SRR3193265 were used for NRI visualization and NRD detection with 1 kb bin resolution as shown in Fig. 2c.
H3K4me1, H3K4me3, H3K9ac, H3K9me3, H3K27ac, H3K27me3 and Polr2a ChIP-seq bam files and peak files, , ChIP input bam files and total RNA-seq bam files of mouse ES cell line Bruce4 were downloaded under accession ENCSR343RKY from https://www.encodeproject.org. DNase-seq bigwig signal file and peak file of mouse ES cell line E14 were downloaded under accession ENCSR000CMW from https://www.encodeproject.org, respectively. ChIP input bam files of mouse ES cell line E14 were downloaded under accession ENCFF224HWC and ENCFF740TUE. Hi-C compartments PC1 values of mouse ES cell was downloaded under accession 4DNESUCLJAZ842 and 4DNESMXBLGKA24 from https://data.4dnucleome.org.

Research involving human participants, their data, or biological material

Policy information about studies with human participants or human data. See also policy information about sex, gender (identity/presentation), and sexual orientation and race, ethnicity and racism.

Reporting on sex and gender	Not involved.
Reporting on race, ethnicity, or other socially relevant groupings	Not involved.
Population characteristics	Not involved.
Recruitment	Not involved.
Ethics oversight	Not involved.

Note that full information on the approval of the study protocol must also be provided in the manuscript.

Field-specific reporting

Please select the one below that is the best fit for your research. If you are not sure, read the appropriate sections before making your selection.

☒ Life sciences ☐ Behavioural & social sciences ☐ Ecological, evolutionary & environmental sciences

For a reference copy of the document with all sections, see nature.com/documents/nr-reporting-summary-flat.pdf

Life sciences study design

All studies must disclose on these points even when the disclosure is negative.

Sample size	No statistical methods were used to predetermine sample sizes. For this study, the sample size usually reflects the number of replicates of a given experiment.
Data exclusions	No data were excluded from the analyses.
Replication	Two independent experiments were performed for H3-wrapping-seq, H4-wrapping-seq, xMNase-wrapping-seq, pulse-chase wrapping-seq and replication-timing sequencing. No replication was performed for time-course MNase-seq and cell-cycle MNase-seq.
Randomization	All samples used in this study were collected without biased selection.
Blinding	For wrapping-seq and replication timing profiling, blinding was not feasible due to the nature of the assessment.

Reporting for specific materials, systems and methods

We require information from authors about some types of materials, experimental systems and methods used in many studies. Here, indicate whether each material, system or method listed is relevant to your study. If you are not sure if a list item applies to your research, read the appropriate section before selecting a response.

Materials & experimental systems

n/a	Involved in the study
☐	☒ Antibodies
☐	☒ Eukaryotic cell lines
☒	☐ Palaeontology and archaeology
☒	☐ Animals and other organisms
☒	☐ Clinical data
☒	☐ Dual use research of concern
☒	☐ Plants

Methods

n/a	Involved in the study
☐	☒ ChIP-seq
☒	☐ Flow cytometry
☒	☐ MRI-based neuroimaging

Antibodies

Antibodies used: H3 antibody, Abcam, ab1791
H4 antibody, Cell Signaling Technology, 14149S

Validation: H3 antibody, validated by manufacture and publications.
H4 antibody, validated by manufacture and publications.

Eukaryotic cell lines

Policy information about cell lines and Sex and Gender in Research

Cell line source(s): Mouse ES cell line (R1) was a gift from Dr. Guohong Li, Institute of Biophysics, CAS.

Authentication: Authentication was based on communication with the providers.

Mycoplasma contamination: The cell line used was tested negative for mycoplasma.

Commonly misidentified lines (See ICLAC register): Not applicable.

Plants

Seed stocks: Not involved.

Novel plant genotypes: Not involved.

Authentication: Not involved.

ChIP-seq

Data deposition

☒ Confirm that both raw and final processed data have been deposited in a public database such as GEO.

☒ Confirm that you have deposited or provided access to graph files (e.g. BED files) for the called peaks.

Data access links
May remain private before publication.

To review GEO accession GSE243091:
Go to https://www.ncbi.nlm.nih.gov/geo/query/acc.cgi?acc=GSE243091
Enter token yfyvkqoafbabtex into the box.

Files in database submission

H3.wrap-seq.rep1-1.R1.fastq.gz
H3.wrap-seq.rep1-1.R2.fastq.gz
H3.wrap-seq.rep1-2.R1.fastq.gz
H3.wrap-seq.rep1-2.R2.fastq.gz
H3.wrap-seq.rep2-1.R1.fastq.gz

H3.wrap-seq.rep2-1.R2.fastq.gz
H3.wrap-seq.rep2-2.R1.fastq.gz
H3.wrap-seq.rep2-2.R2.fastq.gz
H4.wrap-seq.rep1.R1.fastq.gz
H4.wrap-seq.rep1.R2.fastq.gz
H4.wrap-seq.rep2.R1.fastq.gz
H4.wrap-seq.rep2.R2.fastq.gz
MNase-input.wrap-seq.rep1.R1.fastq.gz
MNase-input.wrap-seq.rep1.R2.fastq.gz
MNase-input.wrap-seq.rep2.R1.fastq.gz
MNase-input.wrap-seq.rep2.R2.fastq.gz
MNase.10min.R1.fastq.gz
MNase.10min.R2.fastq.gz
MNase.20min.R1.fastq.gz
MNase.20min.R2.fastq.gz
MNase.30min.R1.fastq.gz
MNase.30min.R2.fastq.gz
MNase.40min.R1.fastq.gz
MNase.40min.R2.fastq.gz
MNase.50min.R1.fastq.gz
MNase.50min.R2.fastq.gz
cellcycle.G1.MNase.R1.fastq.gz
cellcycle.G1.MNase.R2.fastq.gz
cellcycle.G1.sonication.R1.fastq.gz
cellcycle.G1.sonication.R2.fastq.gz
cellcycle.G2M.MNase.R1.fastq.gz
cellcycle.G2M.MNase.R2.fastq.gz
cellcycle.G2M.sonication.R1.fastq.gz
cellcycle.G2M.sonication.R2.fastq.gz
cellcycle.S.MNase.R1.fastq.gz
cellcycle.S.MNase.R2.fastq.gz
cellcycle.S.sonication.R1.fastq.gz
cellcycle.S.sonication.R2.fastq.gz
nascent.dmso.input.rep1.R1.fastq.gz
nascent.dmso.input.rep1.R2.fastq.gz
nascent.dmso.pull.down.rep1.R1.fastq.gz
nascent.dmso.pull.down.rep1.R2.fastq.gz
nascent.tpl.input.rep1.R1.fastq.gz
nascent.tpl.input.rep1.R2.fastq.gz
nascent.tpl.pull.down.rep1.R1.fastq.gz
nascent.tpl.pull.down.rep1.R2.fastq.gz
chase.60min.dmso.input.rep1.R1.fastq.gz
chase.60min.dmso.input.rep1.R2.fastq.gz
chase.60min.dmso.pull.down.rep1.R1.fastq.gz
chase.60min.dmso.pull.down.rep1.R2.fastq.gz
chase.60min.tpl.input.rep1.R1.fastq.gz
chase.60min.tpl.input.rep1.R2.fastq.gz
chase.60min.tpl.pull.down.rep1.R1.fastq.gz
chase.60min.tpl.pull.down.rep1.R2.fastq.gz
mESC.RT-S1.rep1.R1.fastq.gz
mESC.RT-S1.rep1.R2.fastq.gz
mESC.RT-S1.rep2.R1.fastq.gz
mESC.RT-S1.rep2.R2.fastq.gz
mESC.RT-S2.rep1.R1.fastq.gz
mESC.RT-S2.rep1.R2.fastq.gz
mESC.RT-S2.rep2.R1.fastq.gz
mESC.RT-S2.rep2.R2.fastq.gz
mESC.RT-S3.rep1.R1.fastq.gz
mESC.RT-S3.rep1.R2.fastq.gz
mESC.RT-S3.rep2.R1.fastq.gz
mESC.RT-S3.rep2.R2.fastq.gz
mESC.RT-S4.rep1.R1.fastq.gz
mESC.RT-S4.rep1.R2.fastq.gz
mESC.RT-S4.rep2.R1.fastq.gz
mESC.RT-S4.rep2.R2.fastq.gz
WT-H3.mm10.100kb.nri140bp.bdg
WT-H3.mm10.100kb.nrs140bp.bdg
WT-H3.mm10.100kb.nri140bp.NRDs.bed
WT-H4.mm10.100kb.nri140bp.bdg
WT-H4.mm10.100kb.nrs140bp.bdg
WT-input.mm10.100kb.nri140bp.bdg
WT-input.mm10.100kb.nrs140bp.bdg
mESC.RT.smoothed.rep1.bdg
mESC.RT.smoothed.rep2.bdg

mESC.RT.domains.bed

Genome browser session (e.g. UCSC)

Not used.

Methodology

Replicates

Two independent experiments were performed for H3-wrapping-seq, H4-wrapping-seq, xMNase-wrapping-seq, pulse-chase wrapping-seq and replication-timing sequencing. No replication was performed for time-course MNase-seq and cell-cycle MNase-seq.

Sequencing depth

H3.wrap-seq.rep1-1, pair end, read number: 29882953, read length: 150 bp
H3.wrap-seq.rep1-2, pair end, read number: 117962128, read length: 150 bp
H3.wrap-seq.rep2-1, pair end, read number: 25507064, read length: 150 bp
H3.wrap-seq.rep2-2, pair end, read number: 101818518, read length: 150 bp
H4.wrap-seq.rep1, pair end, read number: 30608338, read length: 150 bp
H4.wrap-seq.rep2, pair end, read number: 64387323, read length: 150 bp
MNase.10min, pair end, read number: 58073983, read length: 150 bp
MNase.20min, pair end, read number: 37064701, read length: 150 bp
MNase.30min, pair end, read number: 49282615, read length: 150 bp
MNase.40min, pair end, read number: 64719170, read length: 150 bp
MNase.50min, pair end, read number: 56897262, read length: 150 bp
MNase-input.wrap-seq.rep1, pair end, read number: 11432826, read length: 150 bp
MNase-input.wrap-seq.rep2, pair end, read number: 20002060, read length: 150 bp
cellcycle.G1.MNase, pair end, read number: 33139402, read length: 150 bp
cellcycle.G1.sonication, pair end, read number: 38268776, read length: 150 bp
cellcycle.G2M.MNase, pair end, read number: 30444318, read length: 150 bp
cellcycle.G2M.sonication, pair end, read number: 38786312, read length: 150 bp
cellcycle.S.MNase, pair end, read number: 25911962, read length: 150 bp
cellcycle.S.sonication, pair end, read number: 26404994, read length: 150 bp
chase.60min.dmso.input.rep1, pair end, read number: 40273469, read length: 150 bp
chase.60min.dmso.pull.down.rep1, pair end, read number: 33591975, read length: 150 bp
chase.60min.tpl.input.rep1, pair end, read number: 190858056, read length: 150 bp
chase.60min.tpl.pull.down.rep1, pair end, read number: 47714514, read length: 150 bp
nascent.dmso.input.rep1, pair end, read number: 41022293, read length: 150 bp
nascent.dmso.pull.down.rep1, pair end, read number: 32035590, read length: 150 bp
nascent.tpl.input.rep1, pair end, read number: 43715337, read length: 150 bp
nascent.tpl.pull.down.rep1, pair end, read number: 34643445, read length: 150 bp
mESC.RT-S1.rep1, pair end, read number: 19155587, read length: 150 bp
mESC.RT-S1.rep2, pair end, read number: 31656453, read length: 150 bp
mESC.RT-S2.rep1, pair end, read number: 18079226, read length: 150 bp
mESC.RT-S2.rep2, pair end, read number: 42551417, read length: 150 bp
mESC.RT-S3.rep1, pair end, read number: 20544828, read length: 150 bp
mESC.RT-S3.rep2, pair end, read number: 41344209, read length: 150 bp
mESC.RT-S4.rep1, pair end, read number: 18087794, read length: 150 bp
mESC.RT-S4.rep2, pair end, read number: 27990613, read length: 150 bp
R1-EdU-16hr-xMNase.rep1, pair end, read number: 43748962, read length: 150 bp
R1-EdU-16hr-xMNase.rep2, pair end, read number: 33796229, read length: 150 bp
R1-non-lable-xMNase.rep1, pair end, read number: 46648508, read length: 150 bp
R1-non-lable-xMNase.rep2, pair end, read number: 45540153, read length: 150 bp

Antibodies

H3 antibody, Abcam, ab1791
H4 antibody, Cell Signaling Technology, 14149S

Peak calling parameters

Paired-end reads were trimmed for adaptor sequence using cutadapt v4.3 with parameters: -a AGATCGGAAGAGCACACGTCTGAACTCCAGTCAC -A AGATCGGAAGAGCGTCGTGTAGGGAAAGAGTGT -e 0.1 -n 2 -m 35 -q 30 –pairfilter = any, and then mapped to mm10 using Bowtie2 v2.5.1 with parameters: -I 10 -X 1000 -3 5 --local --no-mixed --no-discordant --no-unal. Duplicates were marked using picard MarkDuplicates v2.27.5 (https://broadinstitute.github.io/picard/) with default parameters and removed using samtools view v1.17 with parameters: -f 2 -F 1024 -q 10. Unique pair-end reads in bam format was converted to bed format. FindHiCCompartments from homer2 was used to detect tight NRD (TiNRD) based on the 10 bins smoothed 100 kb bin. NRI (140bp) data of H3 with default parameter; and the parameter "-opp" was used to output the loose NRD (LoNRD).

Data quality

Reads with high quality were retained using Cutadapt. Unique reads were used for peak calling with mapq > 10.

Software

Cutadapt (version 4.3), Bowtie2 (version 2.5.1), picard MarkDuplicates (version v2.27.5), samtools (version 1.17), bedtools (version 2.30.0), Homer2, Deeptools (version 3.5.1), R (version 4.0.2) and IGV(Integrative Genomics Viewer) (version 2.8.9).
